# Drug-coated balloon vs drug-eluting stent in de novo coronary lesions: a propensity score matched cohort study

**DOI:** 10.1007/s00392-025-02700-w

**Published:** 2025-06-23

**Authors:** Ulrike Baumer, Eva Steinacher, Andreas Hammer, Niema Kazem, Felix Hofer, Bernhard Frey, Irene Lang, Christian Hengstenberg, Rayyan Hemetsberger, Patrick Sulzgruber, Alexander Niessner, Lorenz Koller

**Affiliations:** 1 Division of Cardiology, Department of Internal Medicine II, Medical University of Vienna, Waehringer Guertel 18-20, 1090 Vienna, Austria; 2 Vienna Healthcare Group, 2nd Department of Medicine with Cardiology and Intensive Care Medicine, Clinic Landstrasse, Medical University of Vienna, Juchgasse 25, 1030 Vienna, Austria

**Keywords:** Percutaneous coronary intervention, Drug-coated balloons, Propensity score matching, Drug-eluting stent

## Abstract

**Background:**

The use of drug-coated balloons (DCB) in percutaneous coronary interventions (PCI) is increasing due to potential benefits mainly by avoiding foreign material although a widespread application area beyond in-stent restenosis lacks robust clinical data to date. As such, we aimed to assess the safety and efficacy of DCBs in treating de novo lesions.

**Methods:**

For this analysis, we included all patients treated with DCB in a de novo lesions from 2010 to 2019 at our institution. We performed a 1:1 propensity score matching to pair each DCB intervention with a comparable DES intervention. Follow-up continued until 09/2022 to assess clinical outcomes.

**Results:**

A total of 303 patients with de novo lesion were matched to 303 patients with comparable baseline characteristics. The median follow-up time was 5.7 years (IQR 2.7–9.3). There were no significant differences in cardiovascular (CV) mortality (HR 1.01 [95% CI 0.87–1.19], *p* value 0.874), all-cause mortality (HR 1.05 [95% CI 0.91–1.22], *p* value 0.491), MACE (HR 1.10 [95% CI 0.96–1.26], *p* value 0.170), acute myocardial infarction (HR 1.08 [95% CI 0.90–1.19], *p* value 0.308), or any revascularization (HR 1.03 [95% CI 0.90–1.19], *p* value 0.671) between both groups. However, we observed a trend toward lower rates of target lesion revascularization in patients with small vessel disease (HR 0.84 [95% CI 0.68–1.02], *p* value 0.072), and in side branch lesions (HR 0.79 [95% CI 0.58–1.04], *p* value 0.096).

**Conclusion:**

DCBs demonstrated long-term safety and efficacy in de novo lesions, with promising trends in reducing target lesion revascularization in small vessel disease and side branches.

**Graphical abstract:**

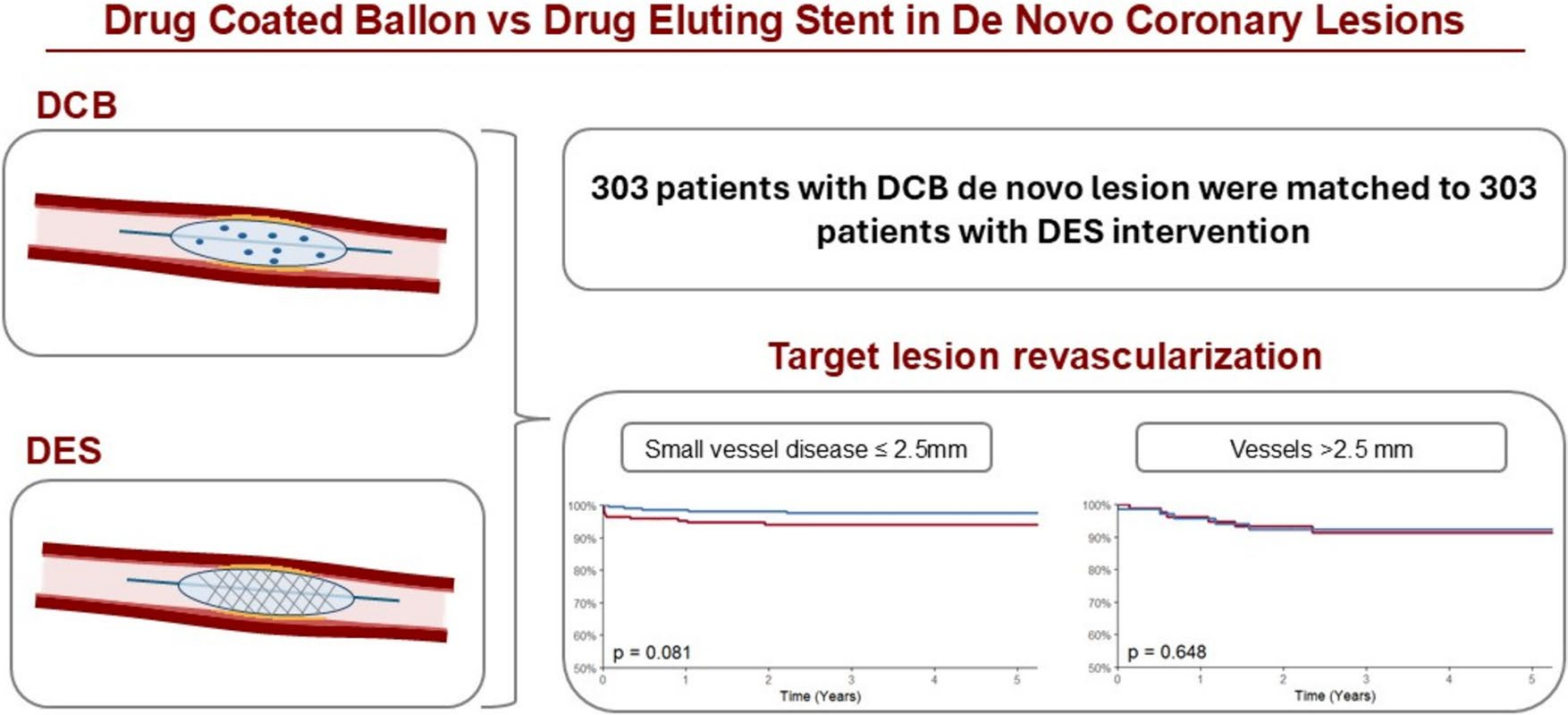

## Introduction

Drug eluting stents (DES) revolutionized the treatment of patients with acute and chronic coronary syndromes and substantially contributed to the success of percutaneous coronary interventions (PCI). Despite well-investigated advantages, their use is also associated with several adverse events, including stent thrombosis and in-stent restenosis caused by impaired re-endothelialization, stent malapposition, hypersensitivity reactions and neoatherosclerosis [1]. The incidence of stent thrombosis ranges from 1 to 2%, remaining a serious and potentially fatal complication in PCI [2]. In-stent restenosis leading to re-intervention still occurs in up to 2% of patients per year [3]. In this respect, drug-coated balloons (DCB) are hypothesized to offer various advantages. No foreign material is left within the vessel, DCBs provide a homogenous drug delivery to the vessel wall and eliminate the need for polymer use. Additionally, they may allow for reduced intensity and duration of antiplatelet therapy with an according decrease in bleeding risk [4]. Primarily used in in-stent restenosis or smaller vessels, we now see a broader clinical application including larger vessels, multivessel disease and even PCIs of the left main stem and bifurcations [5–9]. Still, only a few randomized controlled trials have been conducted with a rather low number of patients. While some were able to show non-inferiority or superiority regarding angiographic outcomes, others did not reach the goal of non-inferiority. This results in partly inconclusive findings and a lack of long-term outcomes [6, 10, 11]. As such, the aim of our study is to assess the use of DCBs in de novo lesion and to determine subgroup-specific long-term outcomes in small and large vessels, respectively. 

## Methods

### Study population

Patients included in this analysis were part of a registry of Vienna General Hospital (Medical University of Vienna) cath lab, which includes all patients who underwent coronary angiography between January 2010 and December 2021 (*N* = 20,979). Data collected included patient demographics, medical history, echocardiograms, laboratory values, and interventional data. In all patients with DCB intervention, reports and angiograms were manually reviewed by experienced cardiac interventionists. Patients with de novo lesions treated with a DCB, excluding those with left main coronary artery interventions, were matched 1:1 using propensity score matching to patients who had comparable DES interventions. The study protocol complies with the declaration of Helsinki and was approved by the ethics committee of the Medical University of Vienna (EK 1745/2024). Data is available from the corresponding author upon reasonable request.  

### Propensity score matching

We performed a 1:1 nearest neighbor propensity score matching with no caliper set. Matching variables were the diameter of the largest de novo lesion (DCB or DES, as continuous variable [mm]), number of vessels intervened in the current PCI (as nominal variable with 1 = 1 vessel, 2 = 2 or more vessels), current intervention for acute myocardial infarction (as nominal variable), age (as continuous variable [years]), sex (as nominal variable), diabetes mellitus (as nominal variable), and previous left ventricular heart failure (as nominal variable).

### Follow-up and endpoints

The primary study endpoint was target lesion revascularization. Secondary endpoints were CV and all-cause mortality, major adverse cardiac events (MACE, including CV mortality, myocardial infarction and ischemic stroke), acute myocardial infarction, any revascularization, and 30-day stent thrombosis. The cause of death was assessed using data from the national death registry. Revascularization data were collected from the Vienna Healthcare Group hospitalizations database (“Wiener Gesundheitsverbund”) including all hospitalizations in Viennese hospital. Target lesion revascularization was determined by screening catheter lab data from the Medical University of Vienna. Additionally, all revascularizations were reviewed manually for confirmation and to identify cases of stent thrombosis.

### Statistical analysis

Clinical data are reported as median and interquartile range (IQR) for continuous variables and counts and proportions for categorial variables. Group comparisons were assessed using *χ*^2^ test for categorical variables or the Kruskal–Wallis test for continuous variables. The Kaplan–Meier estimator method was used to compare observation times, with the log-rank test applied to compare event rates for endpoints.

Cox regression models were used to assess the impact of DCB vs DES on our clinical endpoints. The multivariable model was adjusted for lesion diameter (as continuous variable in [mm]), age (as continuous variable [years]), current acute myocardial infarction (as nominal variable), previous coronary artery bypass graft (as nominal variable), current number of treated vessels (number of vessels as continuous variable), previous known vessel disease (number of vessels as continuous variable), and previous myocardial infarction (as nominal variable). Results are presented as hazard ratios (HR) per 1 standard deviation and 95% confidence intervals (CI) for continuous variables. A two-sided *p* value of < 0.05 was considered statistically significant. For all statistical analyses, R (version 4.3.2; R Foundation for Statistical Computing, Vienna, Austria) and SPSS (version 29.0.0) were used.

## Results

### Patient and procedural characteristics

A total of 918 patients with DCB interventions were screened for this study. Out of these, 303 had at least one de novo lesion treated with a DCB. We performed a 1:1 propensity score matching to identify a control group of 303 patients with DES intervention (matching details described in methods). Overall, both cohorts were well-matched regarding interventional and patient characteristics (details shown in Table 1). Age, sex, comorbidities, and laboratory values were evenly distributed in both groups. Differences were found in the variable multivessel disease prior to index PCI, which was slightly higher in the DCB group. In patients with a DCB intervention, the vessel diameter of the matched lesion was slightly smaller (2.5 mm [IQR 2.0–2.5 mm] vs 2.5 mm [2.25–2.75 mm], *p* value < 0.001) compared to patients with DES intervention. Meanwhile, the total device length used in DCB interventions (41 mm [IQR 20–66 mm] vs 26 mm [IQR [18–48 mm]) as well as the proportion of side branch interventions (150 [49.5%] vs 93 [30.7%]) was significantly higher in the DCB group (respectively *p* values < 0.001). In total, five different DCBs were used and all had a paclitaxel coating with the SeQuent Please DCB as the most frequently used balloon accounting for nearly 80% of all DCB interventions. For lesion preparation in DCB interventions, balloon dilatation was performed using a semi-compliant balloon in 51% of patients, a non-compliant balloon in 40%, and an additional cutting balloon in 2.6% of patients. Baseline clinical and procedural characteristics are summarized in Table 1.

**Table 1:** Baseline Characteristics

Variable	All patients, *n* = 606	DCB intervention, *n* = 303	DES intervention, *n* = 303	*p* value
Clinical Presentation				
Age, years (median, IQR)	64 (55–72)	64 (56–72)	63 (55–72)	0.879
Sex, female, (*n*, %)	162 (26.7)	78 (25.7)	84 (27.7)	0.646
Bod mass index, kg/m^2^ (median, IQR)	27.4 (24.7–31.0)	27.3 (24.7–30.9)	27.6 (24.7–31.0)	0.791
Comorbidities				
Previous myocardial infarction, (*n*, %)	192 (31.7)	106 (35.0)	86 (28.4)	0.097
Previous CABG, (*n*, %)	62 (10.2)	32 (10.6)	30 (9.9)	0.893
Previous known vessel disease, (median, IQR)	2 (1–3)	2 (2–3)	2 (1–2)	< 0.001
Previous left main disease, (*n*, %)	30 (5.0)	16 (5.3)	14 (4.6)	0.852
Diabetes mellitus, (*n*, %)	188 (31.0)	97 (32.0)	91 (30.0)	0.661
Arterial Hypertension, (*n*, %)	358 (59.1)	175 (57.8)	183 (60.4)	0.563
Hyperlipidemia, (*n*, %)	300 (49.5)	154 (50.8)	146 (48.2)	0.570
Previous left ventricular heart failure, (*n*, %)	66 (10.9)	29 (9.6)	37 (12.2)	0.361
Laboratory measures at admission				
NT-proBNP, pg/mL (median, IQR)	547.1 (150.9–1918.0)	564.6 (191.8–1655.0)	521.8 (124.1–2076.0)	0.578
Creatinine, mg/dL (median, IQR)	1.0 (0.9–1.2)	1.0 (0.9–1.2)	1.0 (0.9–1.3)	0.894
HbA1c, %	6.0 (5.5–7.0)	5.9 (5.4–6.6)	6.2 (5.6–7.3)	0.038
C-reactive protein, mg/dL (median, IQR)	0.4 (0.2–1.0)	0.4 (0.2–1.0)	0.3 (0.1–1.0)	0.874
Intervention				
Mean device diameter, matched vessel, mm, (median, IQR)	2.5 (2.25–2.75)	2.5 (2.0–2.5)	2.5 (2.25–2.75)	< 0.001
Total number of vessels treated (median, IQR)	1 (1–2)	1 (1–2)	1 (1–2)	0.073
Total device length, mm, (median, IQR)	33 (20–56)	41 (20–66)	26 (18–48)	< 0.001
Total number of stents used (median, IQR)	1 (1–2)	1 (0–2)	1 (1–2)	< 0.001
Total number of DCB used (median, IQR)	1 (1–1)	1 (1–1)	–	–
Acute intervention (*n*, %)	255 (42.1)	130 (42.9)	125 (41.3)	0.742
Small device used, matched lesion (≤ 2.5 mm, *n*, %)	451 (74.4)	232 (76.6)	219 (72.3)	0.264
Side branch intervention, matched lesion (*n*, %)	243 (40.1)	150 (49.5)	93 (30.7)	< 0.001
Drug-Coated Balloon (*n*, %)				
SeQuent Please	242 (39.9)	242 (79.9)	–	–
Pantera Lux	40 (6.6)	40 (13.2)	–	–
Agent DCB	24 (4.0)	24 (7.9)	–	–
Restore DEB	2 (0.3)	2 (0.7)	–	–
Prevail Paclitaxel-Coated PTCA Balloon Catheter	1 (0.2)	1 (0.3)	–	–

Baseline characteristics, with *DCB* drug-coated balloon, *DES* drug-eluting stent, *IQR* inter-quartile-range, *CABG* coronary artery bypass graft, *NT-proBNP* N-terminal prohormone of brain natriuretic peptide, *HbA1c* hemoglobin A1c

### Clinical outcomes

**Fig. 1:**
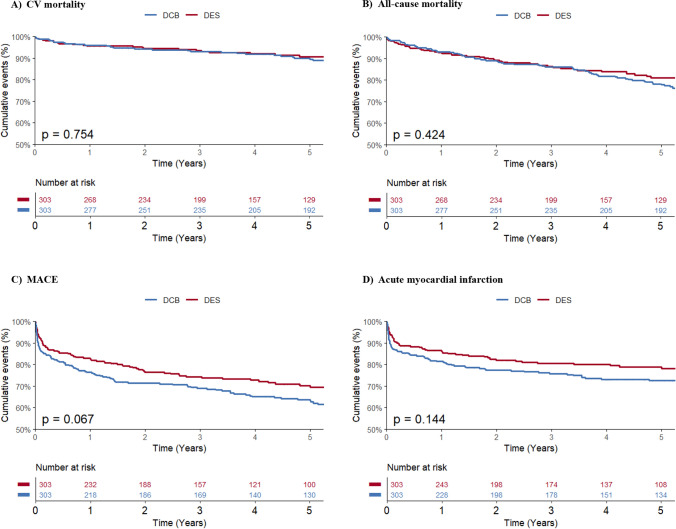
Kaplan–Meier curves and the corresponding 5-year Kaplan–Meier event rates for **A** CV mortality **B** all-cause mortality **C** MACE and **D** acute myocardial infarction comparing DCB and DES. *CV* cardiovascular, *DCB* drug-coated balloon, *DES* drug-eluting stent

Patients were followed up over a median time of 5.7 years (IQR 2.7–9.3). Both groups showed comparable incidence rates of CV mortality (DCB 13.2% [*n* = 40] vs DES 9.9% [*n* = 30], HR 1.01 [95% CI 0.87–1.19], *p* value 0.874), all-cause mortality (DCB 29.7% [*n* = 90] vs DES 21.5% [*n* = 65], HR 1.05 [95% CI 0.91–1.22], *p* value 0.491), MACE (DCB 39.9% [*n* = 121] vs DES 30.4% [*n* = 92], HR 1.10 [95% CI 0.96–1.26], *p* value 0.170), acute myocardial infarction (DCB 27.4% [*n* = 83] vs DES 21.5% [*n* = 65], HR 1.08 [95% CI 0.93–1.25], *p* value 0.308), and any revascularization (DCB 32.0% [*n* = 97] vs DES 25.7% [*n* = 78], HR 1.03 [95% CI 0.90–1.19], *p* value 0.671). The primary endpoint of target lesion revascularization occurred in 32 patients (5.3%), with 12 cases (4%) in the DCB group vs 20 cases (6.6%) in the DES group (HR 0.88 [95% CI 0.75–1.04], *p* value 0.145). Both groups showed very low rates of stent thrombosis within 30 days with 2 cases (0.7%) in the DCB group and 1 case (0.3%) in the DES group. Cardiovascular endpoints are summarized in Table 2. The corresponding 5-year Kaplan–Meier event curves are displayed in Fig. 1.

**Table 2:** Hazard Ratios per 1 standard deviation for clinical and angiographic endpoints

Variable	All patients, *n* = 606	DCB intervention, *n* = 303	DES intervention, *n* = 303	HR per 1 SD (95% CI)	*p* value
CV mortality	70 (11.6)	40 (13.2)	30 (9.9)	1.01 (0.87–1.19)	0.874
All-cause mortality	155 (25.6)	90 (29.7)	65 (21.5)	1.05 (0.91–1.22)	0.491
MACE	213 (35.1)	121 (39.9)	92 (30.4)	1.10 (0.96–1.26)	0.170
Acute myocardial infarction	148 (24.4)	83 (27.4)	65 (21.5)	1.08 (0.93–1.25)	0.308
Any revascularization	175 (28.9)	97 (32.0)	78 (25.7)	1.03 (0.90–1.19)	0.671
Target lesion revascularization	32 (5.3)	12 (4.0)	20 (6.6)	0.88 (0.75–1.04)	0.145
30-day stent thrombosis	3 (0.5)	2 (0.7)	1 (0.3)	-	-

*DCB* drug-coated balloon, *DES* drug-eluting stent, *HR* hazard ratio, *SD* standard deviation, *MACE* major adverse cardiac event

### Subgroup analysis

**Fig. 2:**
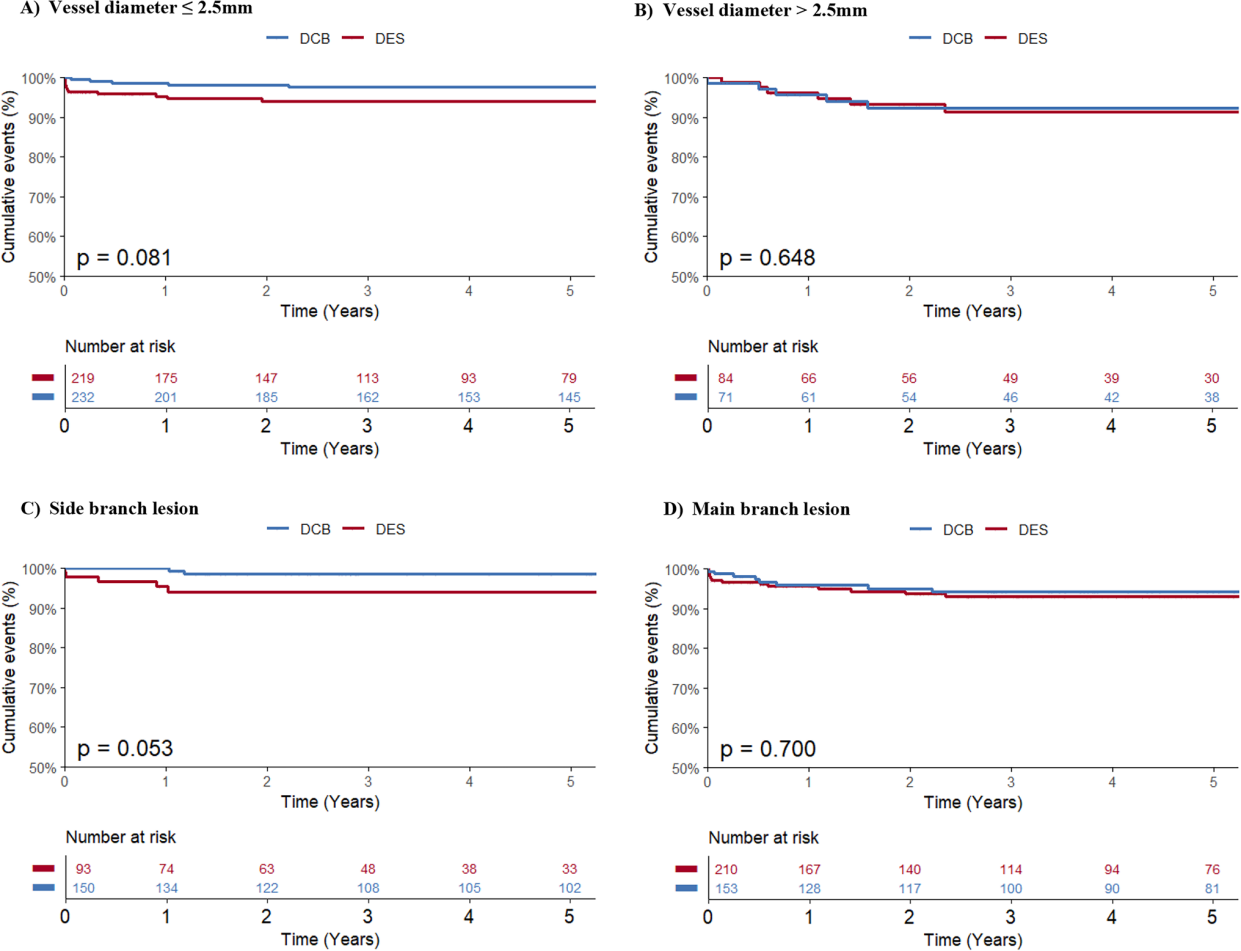
Kaplan–Meier curves and the corresponding 5-year Kaplan–Meier event rates for target lesion revascularization in **A** vessel diameter ≤ 2.5 mm **B** vessel diameter > 2.5 mm **C** side branch lesion **D** main branch lesion. *CV* cardiovascular, *DCB* drug-coated balloon, *DES* drug-eluting stent

Patients were divided into subgroups for vessel diameter (≤ 2.5 mm and > 2.5 mm), side or main branch and acute or elective intervention. When analyzing the interventions by vessel diameter, the data showed that there was a trend to lower rates of target lesion revascularization in DCB interventions in smaller vessels with 2.6% (*n* = 6) in the DCB group vs 5.5% (*n* = 12) in the DES group with an adjusted hazard ratio per 1 standard deviation of 0.84 (95% CI 0.68–1.02, *p* value 0.072). Meanwhile interventions with a diameter > 2.5 mm showed no significant difference (DCB 8.5% [*n* = 6] vs DES 9.5% [*n* = 8], HR 0.95 [95% CI 0.69–1.30], *p* value 0.745). There was a similar trend for target lesion revascularization in side branch interventions, with 1.3% (*n* = 2) in DCB interventions vs 5.4% (*n* = 5, HR 0.79 [IQR 0.58–1.04], *p* value 0.096) in DES interventions (Fig. 2). There was no significant difference in our primary and secondary endpoints when comparing acute and elective interventions. Findings of the subgroup analysis are summarized in Table 3.

**Table 3:** Subgroup analysis – Hazard Ratios per 1 standard deviation

Vessel diameter	≤ 2.5 mm				> 2.5 mm			
	DCB, *n* = 232	DES, *n* = 219	HR per 1 SD	*p* value	DCB, *n* = 71	DES, *n* = 84	HR per 1 SD	*p* value
CV mortality	30 (12.9)	20 (9.1)	0.99 (0.82–1.20)	0.936	10 (14.1)	10 (11.9)	0.95 (0.69–1.31)	0.759
All-cause mortality	65 (28.0)	44 (20.1)	1.0 (0.9–1.2)	0.653	25 (35.2)	21 (25.0)	1.06 (0.80–1.40)	0.679
MACE	90 (38.8)	68 (31.1)	1.03 (0.88–1.21)	0.685	31 (43.7)	24 (28.6)	1.22 (0.94–1.59)	0.136
Acute myocardial infarction	62 (26.7)	44 (22.9)	1.03 (0.87–1.22)	0.726	21 (29.6)	16 (19.0)	1.17 (0.88–1.57)	0.276
Any re-vascular-ization	75 (32.3)	56 (25.6)	1.03 (0.87–1.22)	0.718	22 (31.0)	22 (26.2)	1.04 (0.79–1.37)	0.796
Target lesion re-vascular-ization	6 (2.6)	12 (5.5)	0.84 (0.68–1.02)	0.072	6 (8.5)	8 (9.5)	0.95 (0.69–1.30)	0.745
30-day stent thrombosis	1 (0.4)	1 (0.5)	–	–	1 (1.4)	0 (0.0)	–	–

Side/Main branch	Side branch				Main branch			
	DCB, *n* = 150	DES, *n* = 93	HR per 1 SD	*p* value	DCB, *n* = 138	DES, *n* = 210	HR per 1 SD	*p* value
CV mortality	18 (12.0)	8 (8.6)	0.97 (0.73–1.28)	0.818	22 (15.9)	22 (10.5)	0.98 (0.79–1.21)	0.841
All-cause mortality	33 (22.0)	14 (15.1)	1.01 (0.78–1.32)	0.948	57 (41.3)	51 (24.3)	1.08 (0.90–1.30)	0.408
MACE	58 (38.7)	34 (36.6)	1.97 (0.78–1.22)	0.817	63 (45.7)	58 (27.6)	1.15 (1.97–1.38)	0.114
Acute myocardial infarction	42 (28.0)	26 (28.0)	0.97 (0.78–1.25)	0.908	41 (29.7)	39 (18.6)	1.15 (0.95–1.39)	0.142
Any revascular-ization	48 (32.0)	24 (25.8)	1.01 (0.79–1.28)	0.948	49 (35.5)	54 (25.7)	1.08 (0.90–1.29)	0.433
Target lesion revascular-ization	2 (1.3)	5 (5.4)	0.79 (0.58–1.04)	0.096	10 (7.2)	15 (7.1)	0.98 (0.79–1.21)	0.845
30-day stent thrombosis	0 (0.0)	0 (0.0)	–	–	2 (1.4)	1 (0.5)	–	–

Acute/Elective	Acute				Elective			
	DCB, *n* = 130	DES, *n* = 125	HR per 1 SD	*p* value	DCB, *n* = 178	DES, *n* = 173	HR per 1 SD	*p* value
CV mortality	22 (16.9)	13 (10.4)	1.12 (0.87–1.45)	0.371	18 (10.1)	17 (9.8)	0.94 (0.77–1.17)	0.589
All-cause mortality	37 (28.5)	23 (18.4)	1.14 (0.90–1.44)	0.291	53 (29.8)	42 (24.3)	1.06 (0.88–1.27)	0.570
MACE	62 (47.7)	43 (34.4)	1.14 (0.93–1.39)	0.215	59 (33.1)	49 (28.3)	1.08 (0.90–1.29)	0.424
Acute myocardial infarction	42 (32.3)	31 (24.8)	1.12 (0.90–1.40)	0.317	41 (23.0)	34 (19.7)	1.05 (0.87–1.28)	0.614
Any re-vascular-ization	37 (28.5)	31 (24.8)	0.99 (0.79–1.24)	0.905	60 (33.7)	47 (27.2)	1.06 (0.88–1.28)	0.520
Target lesion revascular-ization	5 (3.8)	5 (4.0)	0.98 (0.75–1.26)	0.851	7 (3.9)	15 (8.7)	0.83 (0.66–1.04)	0.099
30-day stent thrombosis	1 (0.8)	0 (0.0)	–	–	1 (0.6)	1 (0.6)	–	–

*DCB* drug-coated balloon, *DES* drug-eluting stent, *HR* hazard ratio, *SD* standard deviation, *MACE* major adverse cardiac event

## Discussion

In this analysis, we matched 303 patients with a DCB PCI in de novo lesions to patients with DES intervention and analyzed interventional as well as clinical outcomes. DCBs appeared to be a safe and reliable interventional approach compared to DES, with favorable 5-year outcomes regarding target lesion revascularization particularly in small vessels (≤ 2.5 mm) and side branch lesions.

### Small vessel disease

Especially in small vessel disease, a higher rate of restenosis and stent thrombosis is observed with DES-PCI [12, 13]. One of the first large trials, the BASKET SMALL 2 non-inferiority trial, randomized 758 patients with a de novo lesions < 3 mm to either DCB or DES treatment. The data showed that DCB was not inferior to DES regarding the primary endpoint of MACE (7.5% in the DCB group vs 7.3% in the DES group, HR 0.97 [95% CI 0.58–1.64], *p* value 0.918) after 12 months. A three-year follow-up analysis showed safety of DCB over a longer period, with a numerical lower, though not significantly different rate of stent thrombosis in the DCB group [6, 14]. The RESTORE SVD trial compared angiographic outcomes in 230 patients treated with DCB vs second-generation DES for lesions between 2.25 and 2.75 mm. They were able to show non-inferiority in terms of in-segment % diameter stenosis after 9 months (DCB: 29.6 ± 2.0%; DES: 24.1 ± 2.0%; *p* for non-inferiority < 0.001), with comparable rates of target lesion failure (DCB: 4.4% vs DES 2.6%, *p* value 0.72). [15] On the contrary, the recently published REC-CAGEFREE I trial failed to show non-inferiority for DCB regarding the device-oriented composite endpoint, which included CV death, target vessel myocardial infarction, and target lesion revascularization. In this trial, 2272 patients in China were randomized to DCB or DES, regardless of target vessel diameter, and followed up for 24 months. While in the total population, DES showed significant advantages compared to DCBs. Subgroup analysis showed no differences in the primary endpoint for small vessels (all vessel diameters: HR favoring DES 3.04 [95% CI 1.72–5.38], *p* value 0.0001, small vessels: HR 1.17 [95% CI 0.67–2.05], *p* value 0.59, p for interaction 0.02), consistent with other studies focusing on small vessel disease [10]. A meta-analysis of five randomized controlled trials conducted in 2020, which evaluated DCB in small vessel disease, showed lower rates of vessel thrombosis in DCB, alongside similar rates of target vessel revascularization and restenosis. However, DES implantation showed more favorable angiographic outcomes [16]. Another recently published meta-analysis, the ANDROMEDA study by Fezzi et al., compared DCB (paclitaxel-coated) with DES in the treatment of small vessel disease. It included individual patient data from 1154 patients for the primary endpoint (MACE) and 1475 patients for the secondary endpoint (target lesion failure), derived from the BELLO, BASKET SMALL 2 and the PICCOLETO II trials as well as reconstructed data of the RESTORE SVD trial. After three years of follow-up, this study demonstrated significantly lower MACE rates in the DCB group (DCB: 18.5% vs. DES: 24.5%; HR 0.75 [95% CI 0.58–0.96], *p* value 0.022), while target lesion failure rates were similar between the groups (DCB: 14.7% vs DES: 17.6%, HR 0.86 [95% CI 0.64–1.15], *p* value 0.304). [17] Notably, the REC-CAGEFREE I study was not included in either of the meta-analyses. Our data are in line with current trials, demonstrating comparable rates of MACE and revascularizations in de novo lesions though we can see a trend for lower rates of target lesion revascularization in small vessels. With a comparably long follow-up time of 5 years, our data suggest long-term safety of DCB in PCI in those lesions.

### Bifurcation and side branch interventions

The European Society of Cardiology recommends the use of DES in the main branch and a provisional approach in the side branch for most bifurcation stenosis [18]. When comparing DCB to conventional balloon angioplasty for side branch intervention in bifurcations, DCBs appear to offer benefits for patient outcomes. The PEPCAD-BIF trial compared patients with DES in the main branch, randomized to either DCB or conventional balloon angioplasty in the side branch. In patients with angiographic follow-up, restenosis rates were 6% in the DCB group vs 26% in the conventional balloon group after 9 months. There were only 4 cases of target lesion revascularization, three of which occurred in the conventional balloon group [19]. A 2021 study by Li et al. assessed patients with true bifurcation lesions, where the main branch was treated with DES, and patients were randomized to DCB or conventional balloon for the side branch in a provisional stenting strategy. The 12-month follow-up showed a decrease in side branch late lumen loss and lower MACE rates in the DCB group [20]. Smaller single-arm trials have demonstrated that DES in the main branch combined with DCB in the side branch appears to be a safe approach for provisional stenting, including true bifurcation lesions [21, 22]. A small single-arm study involving 49 patients with Medina 0,0,1 lesions showed that DCB-only intervention seems to be a safe option in these patients [23]. However, there are no randomized controlled trials with large case numbers to confirm the benefit of DCB in provisional stenting. With DCB showing promising results in provisional stenting strategies, investigators have started to evaluate DCB-only strategies in bifurcation lesions. Smaller studies have already showed positive outcomes regarding the safety and efficacy of DCB-only in bifurcation lesions [24, 25]. Our data emphasize the long-term safety in both main branch and side branch interventions and even suggest a slight benefit regarding target lesion revascularizations in side branches.

### Paclitaxel versus sirolimus drug-coated balloons

This study only included patients treated with paclitaxel-coated balloon. When examining randomized controlled trials that compare paclitaxel- and sirolimus-coated balloons, findings have been inconclusive [26–28]. A 2024 conducted meta-analysis of five randomized controlled trials including both, in-stent restenosis and de novo lesions, showed no significant outcome difference, except for a larger minimal lumen diameter in paclitaxel treated lesions [29]. Still, larger randomized controlled trials are needed to assess the possible benefits of each balloon type in patient subgroups.

### Limitations

This is a single-center study with its inherent limitations. Although we made various adjustments in our Cox model, residual confounding remains a possibility. Due to the retrospective nature of our study, the individual rationale behind the choice of DCB or DES by various interventionalists could not be reliably reconstructed.

## Conclusion

In our study, DCBs proved to be a safe and effective interventional approach in de novo lesion PCI, offering comparable 5-year outcomes in terms of mortality, MACE and it’s individual components. While in larger vessel diameters, we observed comparable outcomes, and there was a trend for reduced target lesion revascularization in small vessels and side branches treated with DCBs, further supporting the use in these subgroups. Still large randomized controlled trials are urgently needed to assess the use of DCBs in a broad spectrum of interventions including large vessels and more complex interventions and to assess subgroup-specific benefits. 

## Abbreviations

CI: Confidence interval
DCB: Drug-coated balloon
DES: Drug-eluting stent
HR: Hazard ratio
IQR: Inter-quartile range
MACE: Major adverse cardiac event
PCI: Percutaneous coronary intervention
SD: Standard deviation
